# Contribution of T Cell Receptor Alpha and Beta CDR3, MHC Typing, V and J Genes to Peptide Binding Prediction

**DOI:** 10.3389/fimmu.2021.664514

**Published:** 2021-04-26

**Authors:** Ido Springer, Nili Tickotsky, Yoram Louzoun

**Affiliations:** ^1^ Department of Mathematics, Bar-Ilan University, Ramat Gan, Israel; ^2^ Faculty of Life Science, Bar-Ilan University, Ramat Gan, Israel

**Keywords:** TCR - T cell receptor, TCR repertoire analysis, peptide binding, epitope specificity, machine learning, deep learning, long short-term memory (LSTM), autoencoder (AE)

## Abstract

**Introduction:**

Predicting the binding specificity of T Cell Receptors (TCR) to MHC-peptide complexes (pMHCs) is essential for the development of repertoire-based biomarkers. This affinity may be affected by different components of the TCR, the peptide, and the MHC allele. Historically, the main element used in TCR-peptide binding prediction was the Complementarity Determining Region 3 (CDR3) of the beta chain. However, recently the contribution of other components, such as the alpha chain and the other V gene CDRs has been suggested. We use a highly accurate novel deep learning-based TCR-peptide binding predictor to assess the contribution of each component to the binding.

**Methods:**

We have previously developed ERGO-I (pEptide tcR matchinG predictiOn), a sequence-based T-cell receptor (TCR)-peptide binding predictor that employs natural language processing (NLP) -based methods. We improved it to create ERGO-II by adding the CDR3 alpha segment, the MHC typing, V and J genes, and T cell type (CD4+ or CD8+) as to the predictor. We then estimate the contribution of each component to the prediction.

**Results and Discussion:**

ERGO-II provides for the first time high accuracy prediction of TCR-peptide for previously unseen peptides. For most tested peptides and all measures of binding prediction accuracy, the main contribution was from the beta chain CDR3 sequence, followed by the beta chain V and J and the alpha chain, in that order. The MHC allele was the least contributing component. ERGO-II is accessible as a webserver at http://tcr2.cs.biu.ac.il/ and as a standalone code at https://github.com/IdoSpringer/ERGO-II.

## Highlights

A high accuracy TCR-peptide binding predictor discerns the relative impact of T Cell Receptor alpha and beta CDR3, MHC, V and J genes to peptide binding prediction.

## Introduction

T lymphocytes (T cells) are pivotal in the cellular immune response (1, 2) as they recognize specific antigenic peptides bound to major histocompatibility complexes (MHCs) (3, 4). This recognition is governed by the heterodimeric αβ T-cell receptor (2). Within the TCRβ chain, the complementarity-determining region (CDR) 1 and CDR2 loops of the TCR contact the MHC alpha-helices while the hypervariable CDR3 regions interact mainly with the peptide (1, 2). In both TCRα and TCRβ chains, CDR3 loops have the highest sequence diversity and are the principal determinants of receptor binding specificity. The CDR1 and CDR2 are determined by the V gene used in the TCR, while CDR3 is determined both by V, (D in the TCRβ) and J, and by the addition and removal of nucleotides at the VD and DJ junctions (VJ in the alpha chain) (1).

The contribution of the different components of the T-cell receptor and the peptide- bound MHC (pMHC) to the binding has never been fully resolved. Estimating this contribution is important for the prediction of peptide-TCR binding and the design of novel TCRs. We have previously developed ERGO-I (pEptide tcR matchinG predictiOn), a highly specific and generic sequence-based TCR-peptide binding predictor based on novel deep learning methods which utilizes parallel embeddings of TCR and peptides in a joint neural network (5). ERGO-I was only based on the beta chain CDR3 sequence. The prediction accuracy of ERGO-I for the binding of an unseen TCR to a known peptide varied drastically, with the Area Under Curve (AUC) ranging from 0.71 to 0.97. We hypothesized that the difference resulted from the varying relative contribution of the beta chain CDR3 sequence to the TCR-peptide binding prediction accuracy. To test that, we developed ERGO-II characterized by extended embedding that contain other components and tested their contribution to the TCR-pMHC binding prediction accuracy.

Experimentally, our understanding of the TCR repertoires’ diversity, structure, and function has been based on sequencing of the β chain repertoire alone (6), but recent advances in single-cell sequencing have shown that TCR’s antigen specificity is determined by the paired sequences of juxtaposed hypervariable CDR3 regions on both TCR*α* and TCR*β* chains (7). The antigen specificity of each chain was found to be largely dependent on its paired chain, suggesting that both TCRα and TCRβ are needed for the task of TCRs antigenic specificity prediction (6).

Computationally, multiple elements beyond the beta CDR3 have been shown to affect TCR-peptide binding. Glanville et al. (4) in GLIPH, have included CDR3 sequences’ motifs and length, MHC alleles, V-J genes, and clonal expansion level. More recent tools have also used additional components. For examplesTCRbuilder (8) models binding between all TCR CDRs and the peptide, and TCRGP (9) used CDR3 segments from both alpha and beta chains. Alpha CDR3 motifs have, in some cases, played a more dominant role in the selection of anti-viral repertoire than beta CDR3 motifs (10, 11).

Another important element affecting binding is the interaction with the MHC. Conventionally, the Vα and Vβ CDR1 and CDR2 loops, encoded entirely within the germline Vα and Vβ regions, have been thought to predominate the TCRs interaction with the MHC. However, recent structural evidence suggests interactions between the CDR3 region, which predominantly drives antigen specificity, and the MHC (12, 13): Specific amino acids from MHC genes which contact or are spatially proximal to the TCR or peptide in the TCR–peptide–MHC complex have been shown to bias V gene usage (14). Thus, TCR-peptide binding prediction accuracy may be affected by the MHC class and allele. Thus, beyond the TCR beta CDR3 seqeunce (7, 10, 15), ERGO-II also includes T cell type. As both V alpha and beta genes have been shown to bias the naïve CD4+ and CD8+ subsets preference (16, 17), with some segments increasing the odds of being CD4+ (or CD8+) up to five-fold (16).

In the following sections, we describe ERGO-II, show that it obtains a high accuracy in multiple tests, and use it to compute the contribution of each component of the TCR-p-MHC complex to the TCR-peptide binding prediction.

We found that the main contributor is the beta chain CDR3 sequence, followed by the beta chain V and J and the alpha chain, in that order.

## Materials and Methods 

### Model Architecture

**Figure 1:**
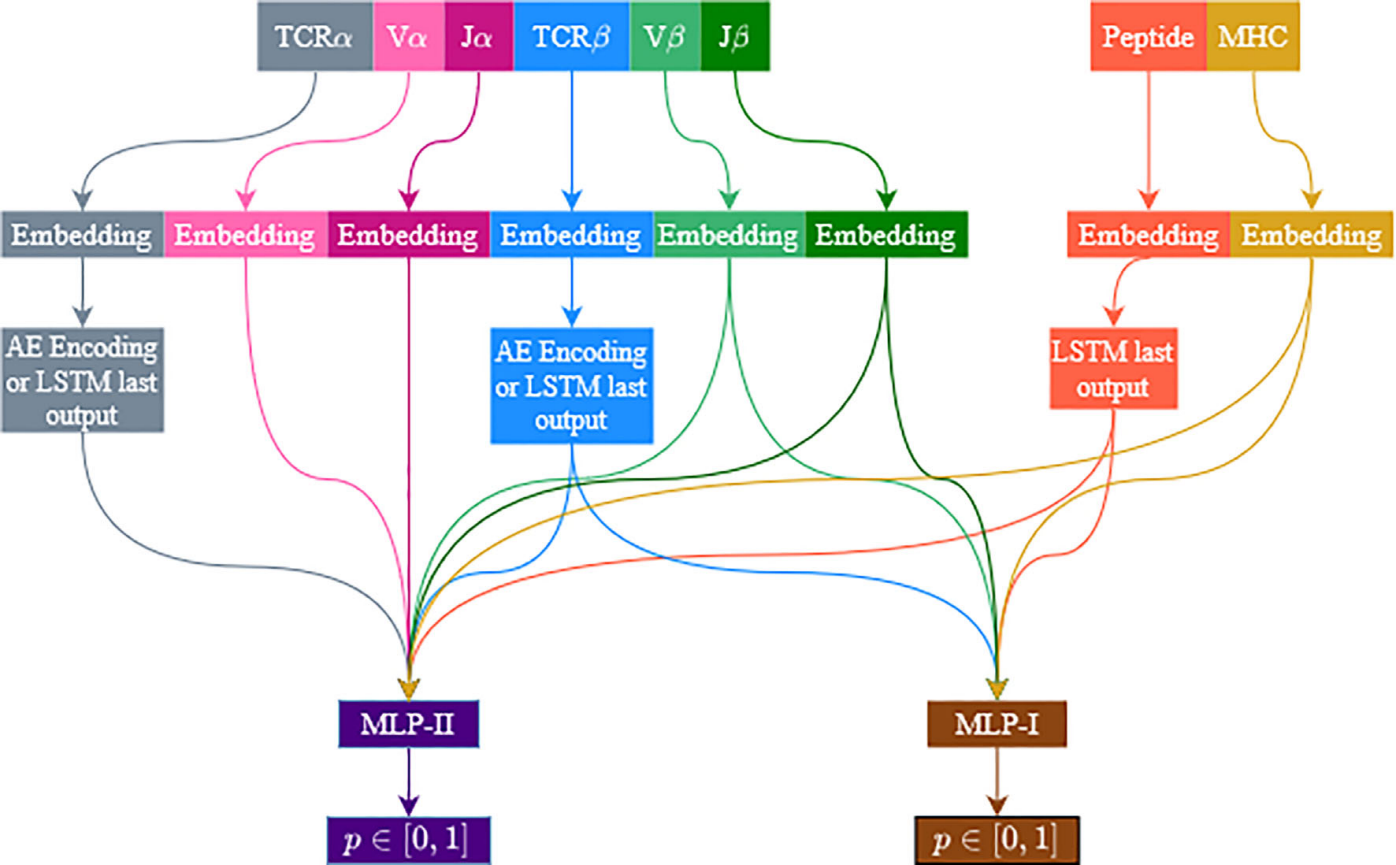
Illustration of the model’s architecture. The architecture is flexible and accommodates several feature configurations. TCRβ and peptide sequences are always used, while V and J genes and the TCRα sequence are optional. Vα and Jα are only used when TCRα is used. MHC usage is optional. TCRs are encoded with an autoencoder or an LSTM. Peptides are always encoded with an LSTM. Other features (except for the T-Cell type which is not illustrated) are encoded using a learned embedding matrix. Two MLP are used, one for samples including TCRα and the other for samples missing the TCRα sequence. We follow the current color code in the following figures.

Similar to ERGO-I (5), ERGO-II is based on the dual encoding of the TCR and the peptide. In the current formalism, additional features are included: the alpha chain, V and J genes, and the T-Cell type are associated with the TCR encoding, while the MHC is associated with the peptide encoding. All features are encoded and concatenated. The encodings are used as the input of a multilayer perceptron (MLP) to predict the binding probability. For the TCR alpha and beta CDR3 sequences, two encoding methods were tested: Long Short Term Memory (LSTM) acceptor encoding and Autoencoder-based encoding. The autoencoder model is pretrained with external TCR data, and its parameters are fine-tuned while training the ERGO-II classifier, while the LSTM parameters are randomly initialized. As explored in ERGO-I, the autoencoder pretrained embeddings are more stable to TCR perturbations. However, in the autoencoder model we limit the length of the TCR input to 28 amino acids (for practical reasons). The peptide is always encoded using the LSTM acceptor method. All other features are encoded using learned embedding matrices, except for the T-Cell type, which is a binary flag (CD4+/CD8+) (**Figure 1**).

### LSTM Acceptor

First, the amino acids were embedded as 10-dimensional vectors using an embedding matrix. Each amino acid was assigned a randomly initialized embedding vector. The TCR or the peptide was fed into an LSTM network as a sequence of vectors. The LSTM network outputs a vector for every prefix of the sequence. The last output was used as the encoding of the whole sequence. We used different embedding matrices and LSTM parameters for the TCRα, TCRβ, and the peptide encodings. The embedding dimension of the amino acids was 10. We use a two-layered stacked LSTM, with 500 units at each layer. A dropout rate of 0.1 was set between the layers.

### TCR Autoencoders

The TCRα autoencoder was trained with TCRα sequences and the TCRβ autoencoder was trained with TCRβ beta sequences. The TCR autoencoders were trained before training the ERGO-II prediction model. The autoencoders’ parameters were then further updated during the ERGO-II model training process. The detailed training and evaluation process and the hyper-parameter configuration of the TCR autoencoder are detailed in the materials and method section of the ERGO-I manuscript (5).

Additional data: The TCR autoencoder was trained on data which was derived from a prospective clinical study (NCT00809276) by Kanakry et al. (18) The dataset is freely available at the Adaptive database (www.adaptivebiotech.com) that provides open access to a variety of datasets of TCRs next-generation sequencing.

### Categorical Features Encoding

The features beyond the CDR3 sequences may be considered as categorical features. We considered the V, J genes, and the MHC allele as categorical features. To encode these features, we used a 50-dimensional embedding vector for each feature. Different embedding matrices were learned for the Vα, Vβ, Jα, Jβ genes and the MHC allele. We did not use an explicit grouping of features so that every unique feature value got a different row in the embedding matrix (e.g. TRAV9 is not the same as TRAV9-3 or TRAV9-1, HLA-A*02:01 and HLA-A*02 have different encodings). We tried using binary encoding by numbering all values and replacing every feature value with its binary representation. However, this led to lower accuracies than the embedding matrix in all tests (results are not reported).

### Dynamic Configuration

Since not all features are always available, ERGO-II follows several configurations, each with different input features, and the architecture of the network depends on which features were used. The TCR sequences are encoded to a vector of 500 dimensions when using LSTM and 100 when using an autoencoder. The V and J genes and the MHC are encoded to a vector of size 50. The peptide encoding dimension is always 500 (we only use the LSTM). All encodings are concatenated, then the concatenated vector is fed into a multilayer perceptron (MLP). Features that were not selected will not contribute to the concatenated vector. The TCRβ chain and the peptide are always selected. V and J genes are selected (or not selected) together. Note that when V and J genes are included, they are included for the chain used (i.e., either only Vβ and Jβ or both Vβ and Jβ and Vα and Jα). **Figure 1** illustrates the model flowchart. Several models were trained to evaluate the contribution of each feature. The same feature combination was used for the training and evaluation phases.

### MLP Classifiers

As mentioned, feature encodings are concatenated and fed into a multilayer perceptron (two MLP networks are trained, see next section). Each MLP contains one hidden layer that contains as many units as the square root of the input vector size. A sigmoid function is applied to the output of the last layer to get a probability value. The activation in the MLP was Leaky ReLU (19). A Dropout rate of 0.1 was set between layers.

### Datasets Used

ERGO-II was trained on two large datasets of binding TCR-peptides (McPAS (20), and VDJdB (21)). As current databases often supply only partial information (The alpha and beta chains CDR3 sequence, their V and J genes, the peptide sequence, the MHC allele and whether the TCR is from a CD4+ or a CD8+ T cell.), ERGO-II is modular, allowing for a combination of different inputs. Each component of either TCR or MHC-peptide was projected into a real vector, using either an autoencoder or a recurrent neural network (implemented as an LSTM (5)). The concatenated elements of each pair were then introduced to a Multi-Layer Perceptron to produce an expected value of 1 for binding pairs and 0 for non-binding pairs. The weight of the classifier, the projection, and the initial embedding were then all trained simultaneously (**Figure 1**).

The datasets studied were updated versions of the databases used to train ERGO-I. McPAS-TCR database (20) was downloaded from http://friedmanlab.weizmann.ac.il/McPAS-TCR/ in July 2020 and VDJdb database (21) was downloaded from https://vdjdb.cdr3.net/ in February 2020. We did not use the 10X Genomics data in the VDJdb database.

### Missing Data

In the datasets studied, we ignored samples that lack TCRβ or peptide sequences. We used two methods to deal with other missing features: For the categorical features (e.g. V, J, MHC) we included an additional symbol for ‘unknown’ in every embedding matrix, so a missing feature value was encoded using the encoding of the unknown symbol. We used the same ‘unknown’ symbol for feature values that appear in the test set but were never seen during training.

For missing TCRα sequences, the unknown flag induces a bias towards the unknown flag. We solved this by using two different MLP networks in the model architecture. One network (MLP-II) deals with samples that contain TCRα, and the other network (MLP-I) deals with samples that have missing TCRα. All other elements were conserved in the presence or absence of the alpha chain. We used the same loss function for both MLP networks. The loss is accumulated over the samples in each batch, and we back-propagate the parameters in the two MLP networks (**Figure 1**).

### Configurations and Hyperparameters Tuning

A Binary Cross-Entropy (BCE) loss was used. We sampled five times more negative examples than positive examples, hence the loss is weighted, respectively, by a factor of 5/6 for positive samples and by 1/6 for negative samples. The optimizer was Adam with a learning rate of 10^-4^ and L2 regularization of 10^-5^. We used batching with a batch size of 128 in training time and 64 in the validation set. An early stopping mechanism was set to stop the training process after three following epochs of decreasing validation Area Under the Curve (AUC) score.

All model hyperparameters were optimized using a grid search in the hyperparameters space. The hyperparameters to optimize were the embedding matrix dimension, the LSTM and Autoencoders dimensions, learning rate, weight decay, activation functions, and dropout rate.

All models were tested with the same grid search. Once the grid search was finished, we chose new sets of training and test and reported their results. Note that the results are quite robust to most parameter changes and that the size of the sample space is much larger than any of the training sets used during parameter tuning. Moreover, the TPP-3 task presented here is performed on peptides never seen in the parameter tuning stage.

All models were implemented with PyTorch, PyTorch-Lightning and Scikit-Learn packages in Python (22, 23).

### Accuracy Tests

Since ERGO-II produces a TCR-pMHC binding prediction and not a single entry classification tasks, multiple tests can be used to assess the prediction accuracy. We followed ERGO (5), and applied some of the tests applied in ERGO-I.

In contrast with most machine learning tasks, where one attempts to predict the output for a given input (e.g. classifying an object), TCR binding is a pairing problem, where one is given a pair of inputs (a peptide and a TCR), and the goal is to predict whether they would bind. As such, there are many ways to divide the train and the test, and as a result many possible tests. We performed four types of tests to assess the accuracy of the pairing prediction (summarized in **Table 1**):

- **Single Peptide Binding – SPB**. Testing whether an unknown TCR binds a predefined target, using (as training information) TCRs known to bind to this target (9, 24, 25). In other words, the target is fixed, and TCRs are divided into disjoint training and test sets. The outcome of such a prediction would be the Area Under Curve (AUC) for the binding of an unseen TCR to this target.
- **TCR-Peptide Pairing I - TPP-I**. Given a large set of peptides and TCRs, test whether a randomly chosen TCR binds a randomly chosen peptide. In this task, all TCR and peptides both belong to training and test sets. However, TCR-peptide pairs are divided into disjoint training and test sets.
- **TCR-Peptide Pairing II - TPP-II** is similar to TPP-I, except that now, TCRs contained in the pairs that belong to the training set cannot belong to the test set.
- **TCR-Peptide Pairing III - TPP-III** is a similar test on pairs, but here neither TCR nor peptide can be in both training and test set.

**Table 1:** Prediction test types.

Method	Test set	Train set	Question answered
SPB Single peptide binding	A TCR not known to bind X	TCRs known to bind peptide X	Does TCR in test bind peptide X?
TPP-I TCR-Peptide Pairing I	Same TCRs and peptides, but differently matched, create binding and non-binding peptides- TCRs pairs that are different from those in train set	Large set of both binding and non-binding peptides- TCRs	Does a randomly chosen TCR bind a randomly chosen peptide?
TPP-II TCR-Peptide Pairing II	TCRs in the training set cannot exist in the test set.		Does a randomly chosen TCR bind a chosen peptide?
TPP-III TCR-Peptide Pairing III	Both TCRs and peptides in the training set cannot exist in the test set.		Does a randomly chosen TCR bind a randomly chosen peptide?

We performed four types of tests to assess the accuracy of the pairing prediction. ERGO-II was trained only on the TPP-I task, and the test was on the different tests above.

ERGO-II was trained only on the TPP-I task, and the test was on the different tests above.

### Data Sampling

The training data was loaded as batches of positive and negative samples. As in ERGO-I, the databases contained only positive samples (paired TCR and peptide), and we created internal negative examples by randomly sampling the data, as further detailed.

To create negative examples, we randomly chose two samples from the data. The TCRβ sequence, TCRα sequence, V, J genes and T-Cell type were taken from the first sample. The peptide and the MHC were taken from the second sample. We merged those feature values into a new negative pair unless of course the pair was known to be positive.

A similar process was applied to create a test set containing both positive and negative examples. The number of negative examples was five times larger than the number of positive examples in both train and test sets (due to more negative sampling). We used 80% of the data for training and the other 20% for evaluation.

The definition of the test data depends on what features were during training: For instance, assuming we have two samples that have the same TCRβ sequence but different TCRα sequences; If we did not use the TCRα data during training, we did not distinguish between those samples, so we did not let the samples appear in both training and test set. However, if we did train the model using the TCRα sequence, these examples were considered different; hence one of them may appear in the test set. Practically, for every experiment reported, we sieved the test set to include only samples that are considered outside the training set, based on the features that were used in the training.

After the regular feature-dependent sieving, the test set was also filtered according to the prediction task. For the SPB task and the TPP-II task, we took only samples containing new TCRβ sequences. For the TPP-III task, we took only samples containing new TCRβ and new peptides.

### Feature Contribution Analysis

**Figure 2:**
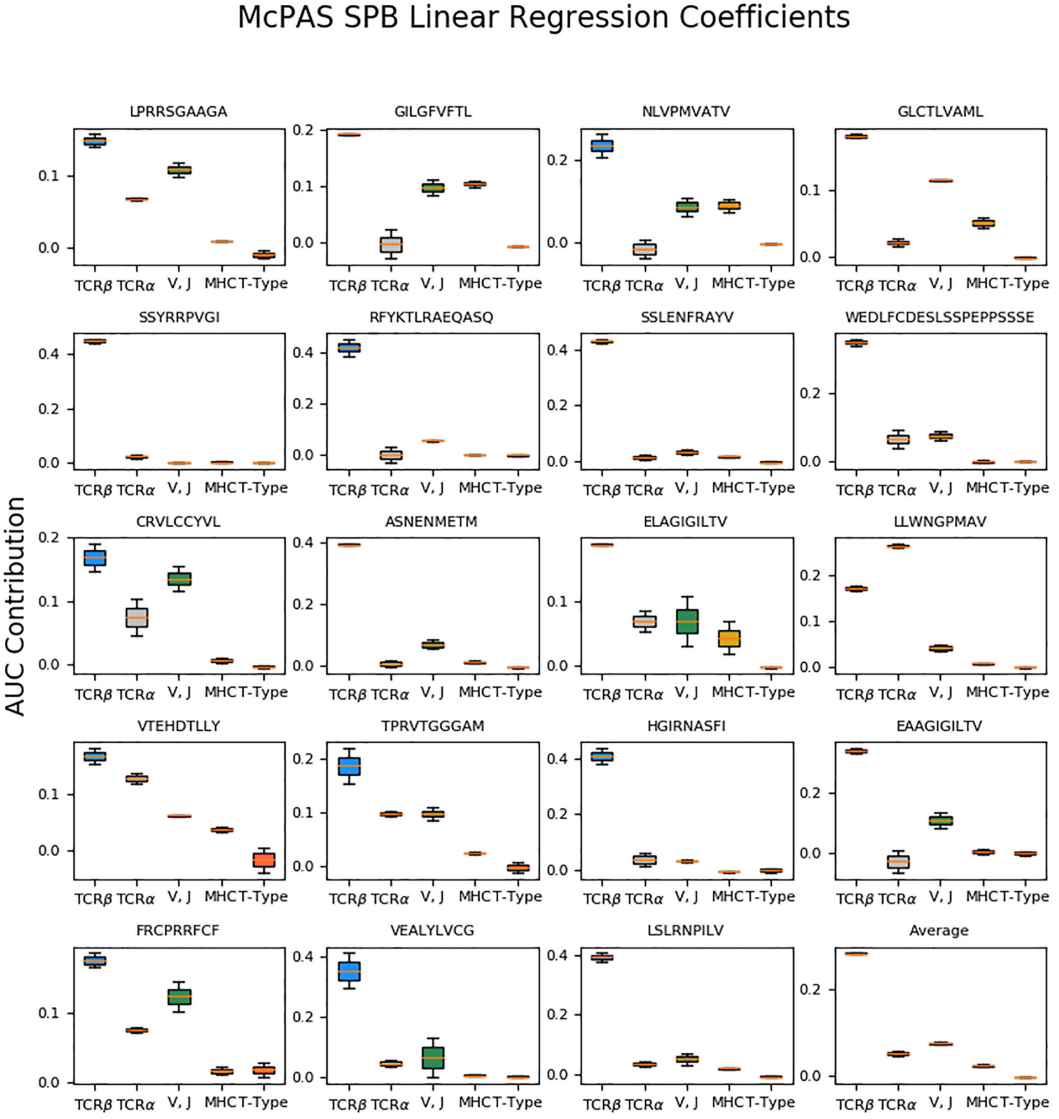
Box plots of the inferred linear regression coefficients of the SPB results on McPAS (20) most frequent 19 peptides, and the average coefficients. The regression is performed on both AE-based and LSTM-based model results. The box size represents the 25^th^ and 75^th^ percentiles over different realizations, and error bars are 10^th^ and 90^th^ percentiles.

We applied ordinary least squares linear regression to fit a coefficient for the contribution of each feature to the AUC. The linear regression coefficient reflected the feature contribution to the validation AUC score. Each feature configuration was represented as a one-hot vector, i.e., in every entry, the value was 1 if the feature was used and 0 otherwise. The analyzed features were TCRα sequence, V and J genes (represented as a single feature), MHC allele, and T-Cell type (CD4+/CD8+). Linear regression was applied to the TPP tasks AUC and the SPB task AUC for the 20 most frequent peptides in the McPAS database. The TCRβ sequence contribution was computed as the constant term in the linear regression minus 0.5, which is the random baseline AUC. When fitting the TPP validation AUC, the test types (TPP-I, TPP-II, and TPP-III) were also represented as a one-hot vector, to infer the effect of the test type on the validation AUC. The reported results, shown in **Figure 2**, are an average of the LSTM-based model coefficients and the autoencoder-based model coefficients.

### Statistical Analysis

Each prediction was performed with a five-fold cross validation. No statistical test was performed on the results, since ERGO-II is sometime better and sometime worse than state of the art methods on the SPB task, and existing methods do not perform the TPP task. In the regression analysis, the confidence interval of the regression are very small. However, they are an underestimate of the variance, since some variance is also due to the difference in machine learning realization. Instead we present the 10, 25, 75 and 90 percentiles of the distribution of the beta values over different learning realizations. In the TPP these variance is very small and is not shown.

## Results

ERGO-II was created by adding the CDR3 alpha segment, the MHC typing, V and J genes, and T cell type (CD4+ or CD8+) to the ERGO-I predictor. ERGO-II is explained in detailed in the methods, but in short, we create two encodings one of peptide-MHC and one for the TCR with the alpha and beta CDR3 sequences and the appropriate V and J genes. The encoders are composed of autoencoders or Long Short Term Memory (LSTM) networks, followed by a Multi-Layer Perceptron (MLP) to produce 1 if the TCR and pMHC bind and 0 otherwise. ERGO contains different MLP for different input configurations (**Figure 1**).

To test the accuracy of ERGO-II, we used two types of tests (See methods). First, we repeated the existing approach, which is to fix the target peptide and predict whether a TCR would bind it (further denoted Single Peptide Binding – SPB). Then, we followed ERGO-I and predicted the random pairing of TCRs and peptides (i.e. pick a TCR and a pMHC and predict whether they would bind- TCR Peptide Pairing -TPP). This last approach is less affected than the SPB by biases in the datasets used in the analysis (**Table 1** and *Methods*).

We first used the most frequent peptides in the McPAS (20) database (a list of these peptides is given in **Supplement Table S1**), computed the test AUC for the two methods of ERGO-II, and compared it with the best results of ERGO-I (**Table 2**). The reported results in **Table 2** are for the five most frequent peptides in both databases. The same peptides were reported in ERGO-I for the SPB task.

**Table 2:** Area Under Curve (AUC) of Single Peptide Binding (SPB) prediction task of the five most frequent peptides in McPAS (20) and VDJdb (21) databases.

Peptide	McPAS			Peptide	VDJdb		
	AE	LSTM	ERGO-I		AE	LSTM	ERGO-I
LPRRSGAAGA	**0.827**	0.820	0.772	KLGGALQAK	**0.765**	0.758	0.731
GILGFVFTL	0.876	**0.886**	0.843	GILGFVFTL	**0.874**	0.864	0.820
NLVPMVATV	0.884	**0.886**	0.835	NLVPMVATV	0.818	**0.827**	0.686
GLCTLVAML	0.861	**0.871**	0.816	AVFDRKSDAK	**0.750**	0.737	0.695
SSYRRPVGI	0.975	0.975	**0.980**	RAKFKQLL	0.820	0.774	**0.828**

The results are for AE-based and LSTM-based models, including TCRα, V, J genes, MHC and T-Cell type data usage. A comparison to ERGO-I best result is shown. Bolded values are the best results on a certain peptide.

The performance of ERGO-II on the SPB test was better than ERGO-I on eight out of ten peptides, and similar on the two remaining peptides In general, the AE performed better than the LSTM, as was also observed in ERGO-I. Note that ERGO-II is trained with more free parameters. Thus, we expect worse results on the train set, when the composition of the data is similar to ERGO-I (i.e. mainly CDR3 sequences of the beta chain).

We repeated the analysis by adding one element of the pMHC-TCR complex at a time: First the beta chain CDR3, then the alpha chain CDR3, then the appropriate V and J gene, followed by the MHC allele, and finally a flag for whether the TCR was a CD4+ or CD8+ receptor (**Table 3**). For all tested peptides, adding information increased the AUC, except for the CD4+/CD8+ flag that for most peptides did not improve the AUC. 

**Table 3:** Area Under Curve (AUC) of Single Peptide Binding (SPB) prediction task of the five most frequent peptides in McPAS (20) database.

Peptide	Model	TCRβ	+TCRα	+ V, J	+ MHC	+ T-cell type
LPRRSGAAGA	AE	0.639	0.704	0.822	**0.830**	0.827
	LSTM	0.656	0.726	0.825	**0.835**	0.820
GILGFVFTL	AE	0.691	0.663	0.773	**0.882**	0.876
	LSTM	0.690	0.713	0.796	**0.894**	0.886
NLVPMVATV	AE	0.761	0.722	0.784	**0.890**	0.884
	LSTM	0.706	0.712	0.820	**0.891**	0.886
GLCTLVAML	AE	0.677	0.704	0.818	**0.862**	0.861
	LSTM	0.683	0.698	0.814	**0.873**	0.871
SSYRRPVGI	AE	0.937	0.968	0.969	0.974	**0.975**
	LSTM	0.952	0.970	0.971	0.974	**0.975**

The results are for AE-based and LSTM-based models. Features in the head of the column are added to the previous features from the left columns. Bolded values are the best results.

To quantitate the contribution of the different components, we computed the AUC using all possible combinations of the different components. We then regressed the AUC on the one-hot representation of the different components. For example, the AUC of a combination of CDR3 beta and CDR3 alpha, but not the V and J information was represented as the sum of the alpha and beta CDR3 contribution to the AUC. This analysis (**Figure 2**), was performed for each of the 19 most frequent peptides in the McPAS (20) database. Since the beta chain CDR3 is always included, we treated the constant coefficient in the regression as the contribution of the beta chain CDR3, and removed from it the expected 0.5 baseline AUC.

For all peptides except for the A*02:01 Yellow Fever LLWNGPMAV peptide, the largest contribution is from the beta chain CDR3 sequence, consistent with the historical focus on this region. The LLWNGPMAV peptide is known to induce a TCR-alpha dependent response (26). None of the added elements had a significant negative effect on the test AUC (single population T-test coefficients vs 0). However, for many peptides, the contribution of elements beyond the beta CDR3 sequence was very small (e.g. SSYRRPVGI, SSLENFRAYV, and HGIRNAFSI). On average, the contribution order followed the one reported in **Table 3** – V and J gene are the leading contributors, followed by the alpha chain CDR3, followed by MHC, and practically no contribution to the cell type.

An exception to that is the allo-HLA reactive Herpes – VTEHDTLLY, where the alpha chain CDR3 is more important than the V gene. This may be due to the binding of this peptide to multiple MHC, and as such to multiple beta chain V genes.

To address the contribution of each component to the TCR-pMHC binding using a peptide independent measure, we used the more complex pairing (TPP) test, where we compute the AUC of pairing a TCR and a pMHC, and tested whether one obtains a high score for properly paired TCR and pMHC and low score for random pairs. As mentioned, we used either known TCR and peptides, but unknown pairing (TPP-I), known peptide with new TCR (TPP-II), and unknown TCR and peptide (TPP-III).

We performed again the sequential addition of features (**Table 4**), with the same results and order of importance as when studying specific peptides. Again adding the T cell type did not improve the AUC. Similarly, as reported in ERGO-I, the McPAS (20) database has a higher AUC than the VDJdb (21) database. Finally, the LSTM had on average a higher AUC than the AE. Note that in contrast with the SPB, the increase in AUC is very large from 0.76-0.78 using only the beta chain CDR3 sequence to 0.93-0.96 with all elements added (except for T cell type). The difference is especially striking in the TPP-II task.

**Table 4:** Area Under Curve (AUC) of TCR-Peptide Pairing (TPP) prediction tasks with either known peptide or TCR (but unknown pairing, TPP-I), known peptide unseen TCR (TPP-II), and unseen peptide and TCR (TPP-III).

Dataset	Features	McPAS		VDJdb (without 10x)	
Model		AE	LSTM	AE	LSTM
Test					
**TPP-I**	**TCRβ**	0.768	0.784	0.736	0.636
	**TCRβ + V, J**	0.909	0.915	0.813	0.799
	**TCRβ + MHC**	0.798	0.806	0.758	0.756
	**TCRβ + T-cell type**	0.858	0.861	0.788	0.787
	**TCRβ + TCRα**	0.831	0.855	0.783	0.800
	**TCRβ + TCRα + V, J**	0.920	0.925	0.833	0.835
	**TCRβ + TCRα + V, J + MHC**	0.936	**0.939**	0.832	0.849
	**TCRβ + TCRα + V, J + MHC + T-cell type**	0.935	0.933	0.862	0.866
**TPP-II**	**TCRβ**	0.756	0.770	0.723	0.625
	**TCRβ + V, J**	0.893	0.900	0.786	0.770
	**TCRβ + MHC**	0.782	0.791	0.737	0.738
	**TCRβ + T-cell type**	0.846	0.852	0.772	0.774
	**TCRβ + TCRα**	0.806	0.832	0.751	0.767
	**TCRβ + TCRα + V, J**	0.903	0.913	0.801	0.805
	**TCRβ + TCRα + V, J + MHC**	0.924	**0.928**	0.800	0.818
	**TCRβ + TCRα + V, J + MHC + T-cell type**	0.922	0.923	0.834	0.840
**TPP-III**	**TCRβ**	0.623	0.652	0.740	0.369
	**TCRβ + V, J**	0.882	0.914	0.944	0.925
	**TCRβ + MHC**	0.595	0.611	0.814	0.777
	**TCRβ + T-cell type**	0.872	0.943	0.833	0.703
	**TCRβ + TCRα**	0.766	0.652	0.666	0.685
	**TCRβ + TCRα + V, J**	0.894	0.900	0.611	0.666
	**TCRβ + TCRα + V, J + MHC**	0.897	0.925	**0.962**	0.833
	**TCRβ + TCRα + V, J + MHC + T-cell type**	0.946	0.927	0.870	0.851

Several feature configurations were examined. The results are the test AUC using either AE-based model or LSTM-based model on McPAS (20) and VDJdb (21) (without 10X Genomics data) databases. Bolded values are the best results on a certain TPP task.

To quantitate the contribution in this test, we again employed a regression on all combinations and all tests. We combined all types of TPP and added a coefficient for each. The main contribution was from the TCR-beta CDR3 sequence, followed by the V and J genes and followed by alpha. However, in contrast with the SPB, here there was a positive contribution of the T cell type, but a negative contribution of the MHC on the test AUC. In other words, the detailed MHC information leads to overfitting on the training set. This may be a limitation of the current encoding, the number of studied pairs, or it may well be that beyond the distinction between classes, the MHC has a very limited contribution.

**Figure 3:**
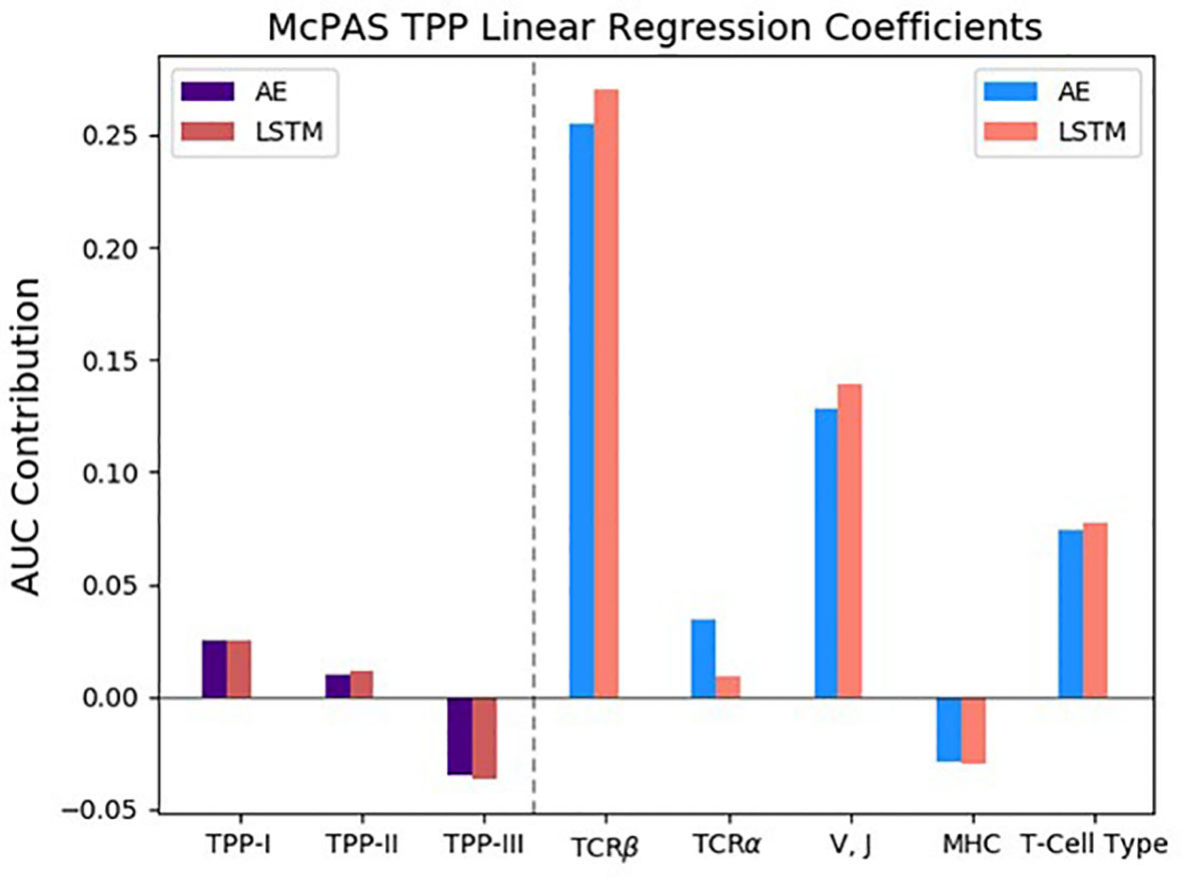
The inferred linear regression coefficients of the TCR Peptide Pairing (TPP) results on the McPAS (20) database, matching the Area Under Curve (AUC) contribution. The included variables in the linear regression were the different types of the TPP evaluations, as well as the various features of ERGO-II. All variables were represented as one-hot vectors according to their usage in the different experiments (see *Methods* and **Table 1**). The left part of the graph shows that the more complex the TPP task, the lower the AUC contribution. Results are reported for AE-based and LSTM-based models. The different models perform similarly and the standard deviation between the models’ inferred coefficients is bounded by 0.013.

As expected, the TPP-I task had a higher AUC than TPP-II, and both contribute more to the AUC than TPP-III (**Figure 3**). However, the difference is limited. Note again that for a setup similar to ERGO-I (only beta chain CDR3 sequence), the results on a test set are worse, since there is more overfitting.

To compare the updated version of ERGO to other state-of-the-art methods, we evaluated the model SPB performance on 22 epitopes from VDJdb database (21) suggested by Jokinen et al. (9), who developed TCRGP. We compared ERGO-II to the mean AUC scores for the VDJdb peptides when using leave-one-out cross-validation as reported by Jokinen et al., when utilizing unique TCR (as defined by CDR3 sequence and V-gene). Jokinen et al. did not include V-genes, and the comparison with their results was performed exclusively on TCR beta CDR3 sequences. We also compared ERGO-II to the TCRex model by Gielis et al. (24), who used a Random Forest algorithm for constructing an epitope-specific classifier. TCRex model includes also V-gene usage. Finally, we compared ERGO-II to Tong et al. (27), who developed the SETE method by applying principal component analysis on the 3-mer TCR CDR3 sequence motifs and training gradient boosting decision tree ensemble learning model. ERGO-II competes with these state-of-the-art results (**Table 5**) on 22 VDJdb peptides. It outperforms all other methods on six peptides and ties with SETE (27) on two peptides. The reported peptides in **Table 5** were taken from Jokinen et al. (TCRGP) paper. All previous methods report their performance on those VDJdb peptides, so we used those peptides for comparison. ERGO-II is highly competitive with other methods. Note that ERGO-II is never trained on any specific peptide. Instead, it is built to predict the binding of any peptide-MHC and TCR pair.

**Table 5:** A comparison of ERGO-II model’s performance to those of other methods on the SPB task.

Peptide	ERGO-II	TCRGP	TCRex	SETE
IPSINVHHY	0.73 ± 0.04	**0.852**	0.84 ± 0.04	**0.85 ± 0.02**
TPRVTGGGAM	**0.91 ± 0.03**	0.892	0.88 ± 0.03	**0.91** ± **0.02**
NLVPMVATV	0.69 ± 0.0	**0.912**	0.72 ± 0.01	0.90 ± 0.01
GLCTLVAML	0.74 ± 0.0	0.926	0.82 ± 0.08	**0.93 + 0.01**
RAKFKQLL	0.75 ± 0.02	0.887	**0.89 ± 0.01**	**0.89 ± 0.02**
YVLDHLIVV	**0.81 ± 0.05**	0.682	0.76 ± 0.07	**0.81 ± 0.04**
GILGFVFTL	0.78 ± 0.0	**0.881**	0.81 ± 0.01	0.87 ± 0.03
PKYVKQNTLKLAT	**0.97 ± 0.01**	0.706	0.72 ± 0.02	0.66 ± 0.06
CINGVCWTV	**0.91 ± 0.02**	0.819	0.75 ± 0.06	0.88 ± 0.05
KLVALGINAV	**0.79 ± 0.04**	0.695	0.73 ± 0.08	0.54 ± 0.03
ATDALMTGY	0.88 ± 0.01	0.678	**0.91 ± 0.04**	0.87 ± 0.04
RPRGEVRFL	**1.0 ± 0.0**	0.801	0.92 ± 0.05	0.93 ± 0.04
LLWNGPMAV	**0.89 ± 0.01**	0.825	0.79 ± 0.01	0.82 ± 0.03
GTSGSPIVNR	**0.92 ± 0.02**	0.864	0.88 ± 0.04	0.72 ± 0.09
GTSGSPIINR	0.84 ± 0.01	0.734	**0.86 ± 0.03**	0.65 ± 0.05
KAFSPEVIPMF	0.88 ± 0.0	0.769	**0.9 ± 0.01**	0.86 ± 0.03
TPQDLNTML	0.92 ± 0.02	0.798	**0.94 ± 0.04**	0.80 ± 0.04
EIYKRWII	0.71 ± 0.01	0.75	0.75 ± 0.06	**0.82 ± 0.05**
KRWIILGLNK	0.81 ± 0.0	0.702	**0.85 ± 0.04**	**0.85 ± 0.02**
FRDYVDRFYKTLRAEQASQE	0.99 ± 0.0	0.893	**1.0 ± 0.0**	0.94 ± 0.04
GPGHKARVL	0.73 ± 0.11	**0.838**	–	0.72 ± 0.04
FLKEKGGL	0.7 ± 0.05	0.817	0.76 ± 0.06	**0.84 ± 0.01**

The reported results show the average and standard deviation of ERGO-II autoencoder and LSTM-based models, using all features. We tested 22 peptides that appear in the VDJdb database (21). ERGO-II is compared to TCRGP (9), TCRex (24) and SETE (27) Classifiers. Reported TCRGP results are for leave-one-out cross-validation when using CDR3 TCRβ. SETE Classifier also uses only TCRβ while TCRex uses TCRβ and Vβ gene. Bolded values are the best AUC results for each peptide. Results with the same average are bolded together.

## Discussion

The initial concept of dual embedding as implemented in ERGO-I had a limited accuracy of predicting whether a previously unseen TCR can bind any peptide from a target protein (TPP task), and a varying accuracy for the binding prediction of new TCR to known peptides (SPB task). We hypothesized that the limited accuracy may result from a lack of essential factors in the analysis, such as the alpha chain or the MHC. To test the relative contribution of different components, we developed ERGO-II, which incorporates both alpha and beta chains, V and J genes, MHC typing, and the cell type for the evaluation of the SPB score of several peptides. We found that for most tested peptides the V and J genes and following it the alpha CDR3 segment contributed significantly to the prediction, while the contribution of the MHC differed between the peptides and cell type contribution was negligible for a known peptide. When predicting binding to unseen pMHC, the MHC information contributed to the prediction at the cell type (i.e., CD4+/CD8+) but we did not detect the influence of the MHC group.

Paired αβ repertoire contains useful information about TCR- MHC recognition, and αβ pairings appear to synergistically drive TCR-MHC interactions (7). Vαβ gene pairings were found to be the TCR most informative feature of T cell lineage, supporting the existence of germline-encoded paired αβ TCR-MHC interaction motifs (7). This is in line with the germline-encoded theory of MHC-TCR restriction, that TCR-MHC bias results from of co-evolution of TCR and MHC genes (28), with evolutionarily conserved “recognition motifs”, i.e., amino acid motifs in the TCRs that are biased for evolutionarily conserved recognition motifs in the MHC molecules (8).

The SPB prediction results on McPAS’s 19 most frequent peptides showed different patterns of the contribution of the alpha, V, J genes and MHC components. With CDR3 beta at the baseline of prediction, as it is considered the most important determinant of binding prediction, different pMHC benefited differently from the other components of the p-MHC-TCR complex. For example: prediction results for LLWNGPMAV yellow fever viral peptide were highly influenced by the alpha CDR3 component. This may be explained by the fact that most data was of paired alpha-beta chains, but also by the immunodominant response to this yellow fever viral epitope, which has been found to be biased for alpha V gene 12-2 (26).

Another peptide with a biased alpha V gene contribution is the melanocytes-associated ELAGIGILTV (29) peptide. Here, the fact that most (> 87%) TCRs that bind this peptide have TRAV12-2 gene (29) may explain the large contribution of the V gene to the SPB predictions. As we had almost no alpha CDR3 data for the wild-type epitope we could not establish a contribution of the alpha CDR3 component on prediction results. However, for the modified MART-1/Melan-A26-35 peptide used to stabilize pMHC binding, EAAGIGILTV, where the natural alanine at position 2 has been modified to leucine (30), we had sufficient data on the alpha CDR3 component and this improved the prediction.

### Study Limitations

Most current algorithms are trained with a pre-defined peptide, and testing for different TCRs whether they bind this peptide. This is very prone to sampling biases, some of which may be biologically relevant – for example, the TRAV12-2 bias towards several epitopes, such as MART-1 and LLWNGPMAV, a yellow-fever epitope. However, other biases such as implicit information leakage from the training set to the test may stem from the fundamental bias in the experimental data that evolved around specific peptide-MHC-TCR triads found in specific pathologies, such as tumor-infiltrating lymphocytes. This presents two challenges: first, unless aggressive dataset curation is done, this will necessarily produce extremely similar sequences in the training and validation datasets, potentially inflating performance. Second, even in cases where certain TCR-pMHC triads are overrepresented, it does not necessarily mean that they are more important. In ERGO-II we have made two main steps to reduce the sampling bias: A) We added a test, where we test the prediction of TCR-pMHC binding for a previously unseen peptide. B) We train over random sets of TCR-pMHC pairs, and not a single peptide. This obviously does not remove all biases, but limits them. Further elaboration is needed to determine the cause for different patterns of contributions of the alpha CDR3, MHC typing, V and J genes found in different peptides and check if all prediction weights are coming from bona fide features of the biology or if some may result from spurious features.

The current analysis was sequence-based but used highly non-linear functions. Such functions can capture indirect links, such as the ones influenced by the secondary structure of the molecules and three-dimensional aspects of the interactions. Still, better structural measures may lead to clearer definitions of the relative contribution of each component.

Our results serve as an important additional step toward the development of predictive tools for biomarker development.

## Data Availability Statement

Publicly available datasets were analyzed in this study. This data can be found here: http://friedmanlab.weizmann.ac.il/McPAS-TCR/ and https://vdjdb.cdr3.net/.

## Author Contributions

IS and YL developed the ERGO-II algorithm. IS implemented the ERGO-II algorithm and the experiments. NT and YL wrote the manuscript. All authors contributed to the article and approved the submitted version.

## Conflict of Interest

The authors declare that the research was conducted in the absence of any commercial or financial relationships that could be construed as a potential conflict of interest.
