# Supplementary material for: Contribution of T Cell Receptor Alpha and Beta CDR3, MHC Typing, V and J Genes to Peptide Binding Prediction
Source: Front Immunol. 2021 Apr 26;12:664514. doi: 10.3389/fimmu.2021.664514 (PMC8107833; doi:10.3389/fimmu.2021.664514)
Supplement: Supplementary file 1 [file Table_1.docx]

| **Database** | **Dataset** | **Number of samples** | **Number of positive samples** | **Unique TCRβ** | **Unique TCRα** | **Unique peptides** | **Unique MHC** |
| --- | --- | --- | --- | --- | --- | --- | --- |
| **McPAS** | **Train** | 69780 | 11630 | 9490 | 2424 | 319 | 70 |
|  | **Test** | 17328 | 2888 | 2577 | 722 | 194 | 58 |
| **VDJdb** | **Train** | 209316 | 34886 | 27231 | 13895 | 200 | 56 |
|  | **Test** | 53154 | 8859 | 7823 | 4167 | 150 | 45 |

**Supp. Mat. Table S1:** Train and test dataset statistics. Notice that most of the samples in a dataset are synthetic negative combination of TCR and a peptide (similar to ERGO-I, see methods). Unique counts are taken within a single dataset. Intersection of some features between train and test datasets is possible (see methods).
